# Multiple electron transporting layers and their excellent properties based on organic solar cell

**DOI:** 10.1038/s41598-017-08613-7

**Published:** 2017-08-29

**Authors:** Ziyan Yang, Ting Zhang, Jingyu Li, Wei Xue, Changfeng Han, Yuanyuan Cheng, Lei Qian, Weiran Cao, Yixing Yang, Song Chen

**Affiliations:** 1 School of Optoelectronics, Beijing Institute of Technology, Beijing, 100081 China; 2 Key Laboratory for Special Functional Materials of Ministry of Education, Henan University, Kaifeng, 475004 Henan China; 3 TCL Corporate Research, Shenzhen, 518052 Guangdong China; 4 College of Chemistry, Chemical Engineering and Materials Science, Soochow University, Suzhou, 215123 Jiangsu China

## Abstract

To improve the performance of inverted polymer solar cells based on a ternary blend of polymerthieno [3,4-b] thiophene/benzodithiophene (PTB7), [6,6]-phenyl C_71_-butyric acid methyl ester (PC_71_BM) and indene-C60-bisadduct (ICBA), a two-layer structure of zinc oxide (ZnO) and Al-doped zinc oxide (AZO) nanoperticles is used to improve electron extraction. Comparing to ZnO, AZO has lower work function and thus provides larger built-in potential across the organic heterojunction, resulting in more efficient photo-current extraction and larger open circuit voltages. Optimum devices with ZnO/AZO nanoparticles show enhancement of both short circuit current and open circuit voltage, leading to a power conversion efficiency (PCE) of 8.85%. The argument of energy level buffering and surface morphology is discussed in the paper. Finally, using a trilayer electron transporting unit of ZnO/AZO/PFN, the interface dipole between the organic active layer and AZO is introduced. The PCE is further enhanced to 9.17%.

## Introduction

Polymer solar cells (PSCs) have attracted tremendous research and industry attentions over the two past decades, due to their promising potential as low-cost, clean and flexible energy sources. PCE of PSCs has been enhanced with the synthesis of new active materials and design of novel device architectures^1–6^. Among those popular photo-active polymers, polythieno [3,4-b]-thiophene-co-benzodithiophene (PTB7) stands out as one of the most studied materials. The PCE of Single-junction devices based on PTB7 and fullerenes has exceeded 10% with advanced device structure and fabrication processes^7–9^.

In PSCs, electron transport layers (ETLs) play a critical role in determining the device performance^10–13^. Oxides with low work function are favored in such case as ETL need to form Ohmic contact with the electron acceptor and provides larger built-in potential and lower series resistance. Among the metal oxides that have been utilized in PSCs as the charge transport layers^14–19^, zinc oxide (ZnO) is one of the most popular material, due to its non-toxicity, high electron mobility, optical transparency, solution processability and the fact that ZnO’s Fermi energy and conduction band minimum match well with the LUMO levels of commonly used acceptor materials^20–24^. In our previous work, ZnO nanoparticle (NP) were utilized for electron transport layers in PSCs and quantum-dot light-emitting diodes, in both of cases the addition of ZnO NPs gives higher device efficiency and better lifetime^25–30^. What makes the material more interesting is that the Fermi energy and carrier concentration of ZnO NPs can be tuned through atomic doping^31^. Krebs al. demonstrated that Al-doped ZnO (AZO) can significantly improve the conductivity of the ZnO layer and thus the device performance^32^. By changing the doping concentration of Al, the energy levels in the conduction band of ZnO NPs can be tuned to better match the LUMO levels of different electron acceptor materials. Therefore, the energy level alignment between ETL and electron accepting fullerenes, as well as the built-in potential of the heterojunction, can be further improved^11–13, 33, 34^. However, high transparency in the long wavelength is depressed due to metal doped in ZnO. Until now, materials or structures that can improve both the light transmittance of the device and the voltage as well as the charge transport of devices using ZnO materials have not been found.

In this work, we report a sol-gel method to synthesize AZO nanoparticles. The synthesized AZO NPs show a lower work function than ZnO NPs. To create favored energy level buffering between ITO and electron accepting fullerenes, we stacked two layers of ZnO and AZO nanoparticles as ETL to improve both the charge transport and light transmittance. The enhancement performance of PTB7:PC_71_BM:ICBA(P:P:I) inverted cells with a two-layer ETL was obtained comparing with that only with ZnO NPs. A larger built-in potential of this two layers structure were also discussed here. An optimum PCE of 8.85% is achieved with optimized doping in AZO, comparing to the control device with only ZnO as ETL and a PCE of 8.34%. A trilayer structured ZnO/AZO/PFN(Poly[9,9-bis(3′-(N,N-dimethyl)-propyl-2,7-fluorene)-alt-2,7-(9,9-dioctylfluorene)]) based on the two structure to further enhance built-in potential of the hetrojunction, was utilized to a PCE of 9.17%. In the meantime, it is also observed that PFN effectively passivates the surface defects of AZO layer^35, 36^.

## Results and Discussion

**Figure 1:**
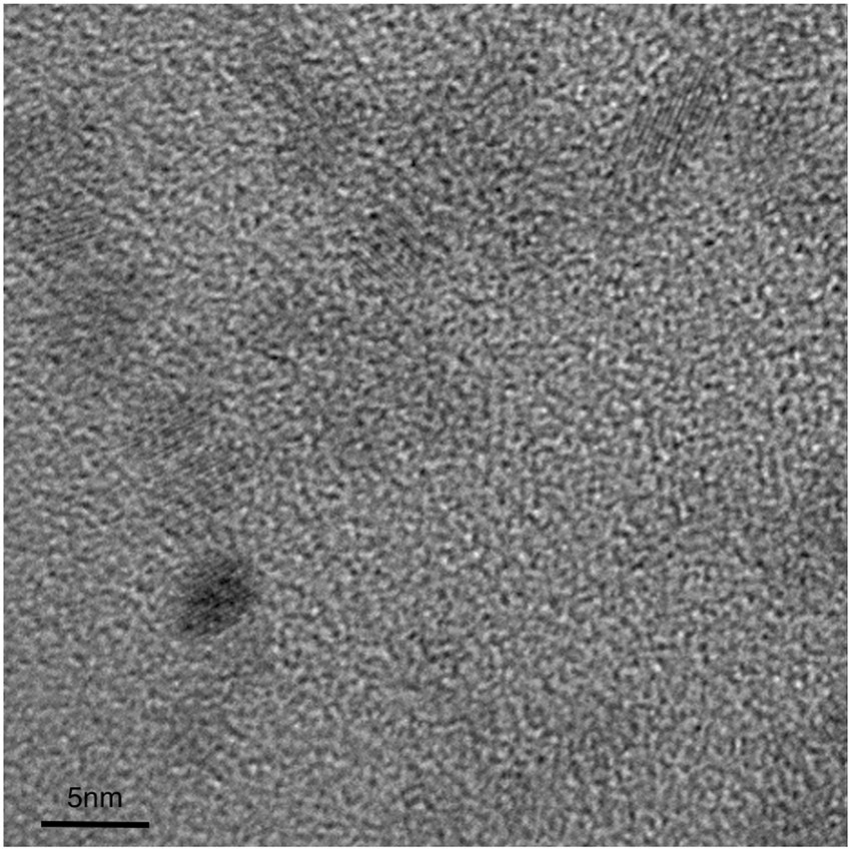
TEM image of AZO.

**Figure 2:**
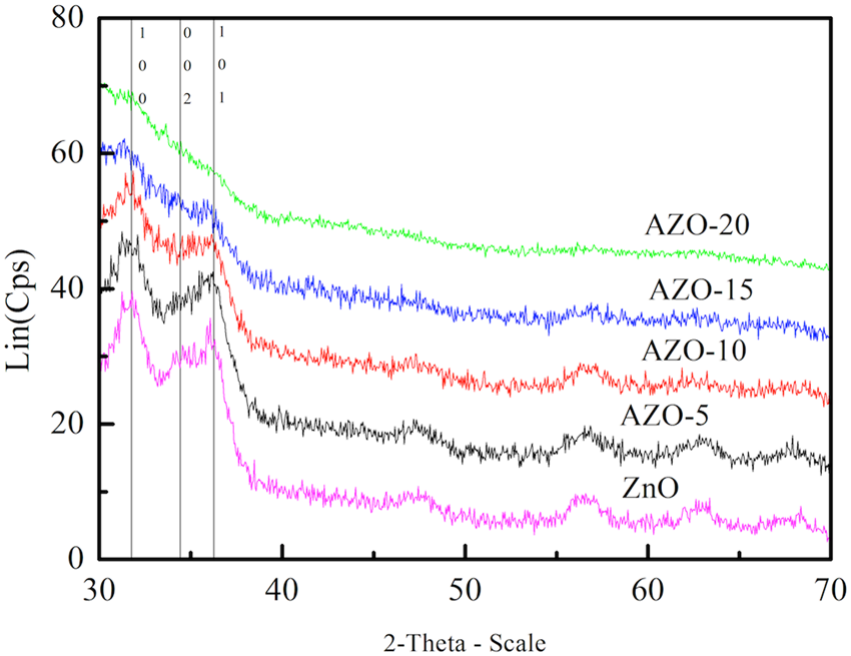
XRD patterns from ZnO (purple line) together with AZO-5, AZO-10, AZO-15, AZO-20 on glass.

ZnO nanoparticles (NPs) were synthesized using the same sol-gel method as our previous reports (see methods for the details)^25^. Al doped ZnO (AZO) NPs were synthesized using similar method, except that the Al precursor (Al(NO_3_)_3_) is mixed together with the Zn precursor (znic acetate) first before the reaction. The doping concentration of AZO NPs is controlled by the Al(NO_3_)_3_-to-zinc acetate ratio. In this report, AZO NPs are synthesized with initial doping concentrations of 2.5%, 5%,10%, 15% and 20%, which is molar ratio(abbreviated as AZO-2.5, AZO-5, AZO-10, AZO-15 and AZO-20, respectively) in precursors. The actual doping concentrations in the synthesized nanoparticles were measured by energy dispersive spectroscopy (EDS). We also confirm the Al contents with X-ray photoelectron spectroscopy (XPS) (Table S2, Figure S4). The EDS detailed result of Zn:Al atomic ratio is listed in Table 1. As shown in the representative transmission electron microscope (TEM) image in Fig. 1, AZO nanoparticles have particle diameters of 3-5 nm which is similar to that of the pure ZnO NPs reported earlier^25^. Shown in Fig. 2 are the X-ray diffraction patterns (XRD) of the ZnO NPs and AZO NPs with different doping concentrations. The ZnO, AZO-5, and AZO-10 NPs share the same diffraction peaks at 2θ = 31.77°, 34.42°, and 36.26° which are typical for crystalline ZnO. Such characteristic peaks of ZnO become less pronounced with the addition of dopant. Eventually, there is no obvious diffraction peaks on the XRD pattern of AZO-20. We attributed such decrease in crystallinity to the radius difference between the Al and Zn ions (R_Al_ = 0.053 nm, R_Zn_ = 0.072 nm)^37^ and the doping of Al ions deteriorate the ZnO crystal structure^38, 39^. Thus our results indicate that Al^3+^ ions are found at interstitial sites, resulting in a decreased lattice parameter of ZnO crystals. Actually, the AZO diffraction peaks has a very little shift when compared with ZnO (in support information Table S3). The success of electrical doping is validated. An electron-only device of AZO shows a ten-fold enhancement of electrical current conduction than the control with undoped ZnO NPs (in support information Table S1, Figure S1).

**Table 1:** EDS data of AZO-x, ZnO.

Element (at%)	O	Zn	Al
AZO-2.5	45.41	53.62	0.97
AZO-5	46.09	52.29	1.62
AZO-10	46.38	51.27	2.35
AZO-15	54.11	41.85	4.04
AZO-20	50.06	42.22	7.72
ZnO	47.83	21.14	/

**Figure 3:**
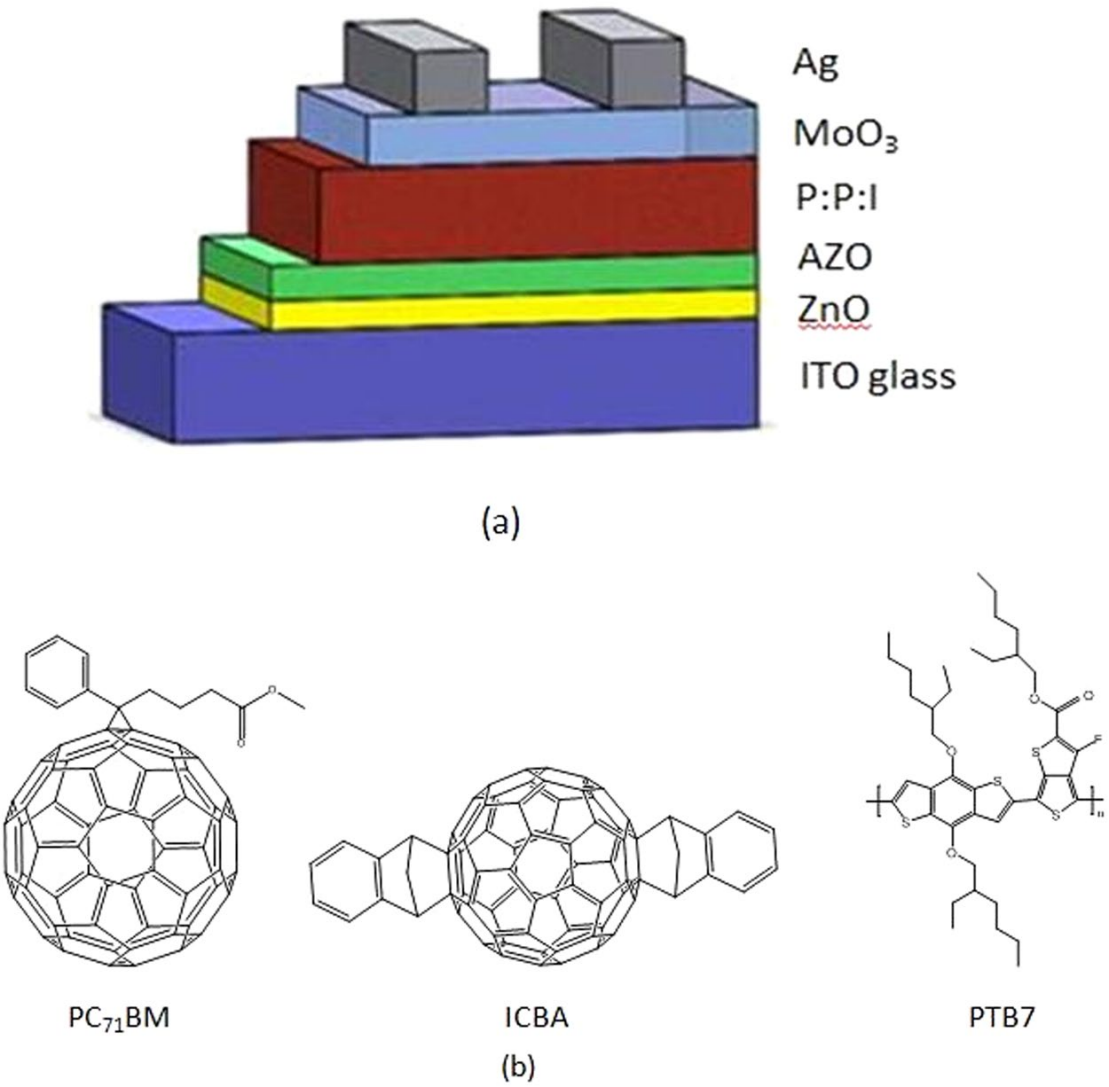
(**a**) the construction of device employed in this work; (**b**) Molecule structures of PTB7, PC_71_BM and ICBA.

**Figure 4:**
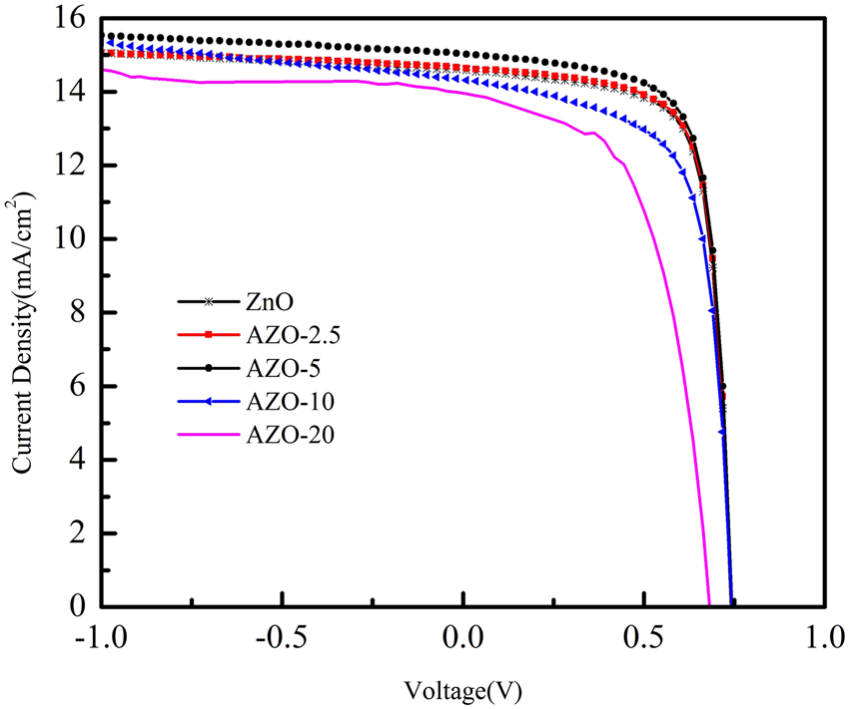
J–V curves of ITO/ZnO(5 nm)/PTB7:PC_71_BM:ICBA/MoO_3_/Ag and ITO/AZO-x (x: 2.5,5,10,20; 5 nm)/PTB7: PC_71_BM:ICBA/MoO_3_/Ag devices.

**Figure 5:**
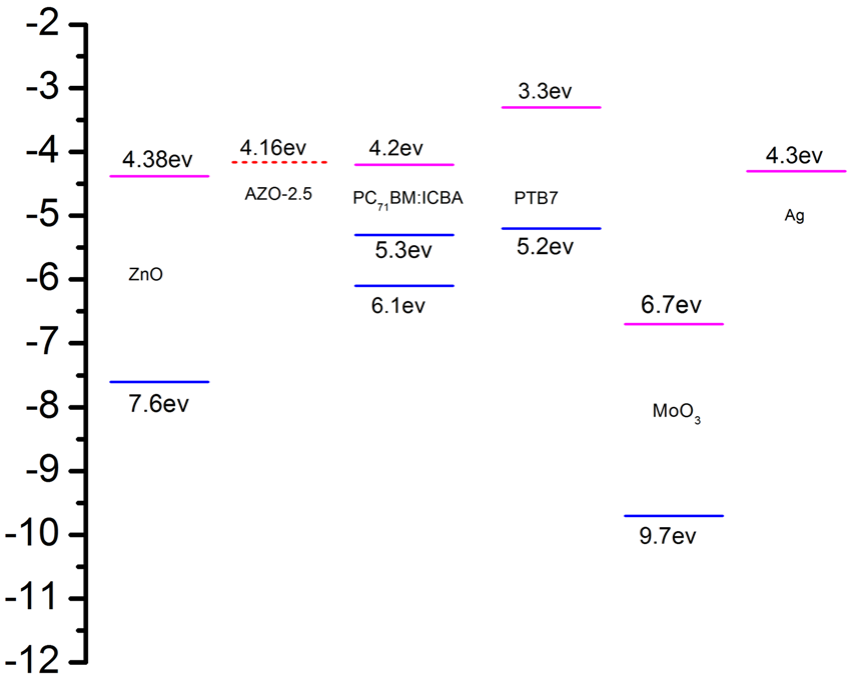
Device energy levels of the inverted-type PSCs ICBA.

Figure 3(a) schematically shows the device architecture of a PTB7:PC_71_BM: ICBA device with ZnO NPs or AZO NPs as electron transport layers, where in a 105 nm-thick PTB7:PC_71_BM:ICBA active layer is sandwiched between the electrodes and electron/hole transporting layers^40–42^. Such a ternary blend is adopted for the enhancement of open circuit voltages. The molecule structures of PTB7, PC_71_BM and ICBA are shown in Fig. 3(b). Shown in Fig. 4 are the current density-voltage (J-V) characteristics of the PTB7: PC_71_BM: ICBA devices with ZnO, AZO-2.5, AZO-5, AZO-10, and AZO-20 NPs as electron transport/selective layers under simulated AM 1.5 G illumination. The device performance slightly improved for the devices with AZO layer with increased Al doping concentration. The open circuit voltage (Voc) of the devices increases from 0.741 V to 0.742 V and to 0.744 V and the short-circuit current density (Jsc) increases from 15.2 mA/cm^2^ to 15.3 mA/cm^2^ and to 15.6 mA/cm^2^ when utilized the AZO layers with increased Al doping concentration from 0 (ZnO NPs) to 2.5% (AZO-2.5 NPs) and to 5% (AZO-5 NPs). Though the device with AZO-5 layer has a relatively lower fill factor (FF) of 72.9% compared to that of 73.4% and 73.6% for the devices with ZnO and AZO-2.5, it shows a higher PCE of 8.56% than that for the devices with ZnO and AZO-2.5 layers (8.34% and 8.41%). We attribute such improvement to the low work function of AZO which increases the built-in potential of the hetrojunction. The energy level of AZO layers with different Al doping concentration was measured with Kelvin Probe technique. As shown in Fig. 5, the undoped ZnO NPs film has a work function of 4.38 eV, and the value of work function decreases to 4.16 eV with a doping of 2.5% Al (AZO-2.5). Compared that of the undoped ZnO NPs, the work function of AZO-2.5 is closer to the LUMO level of PC_71_BM:ICBA (4.2 eV)^5, 43^, leading to larger built-in potential across the organic heterojunction and thus better charge transport/extraction. Therefore, simultaneous enhancement of Jsc and Voc is achieved in inverted cells using AZO is ETL.

**Figure 6:**
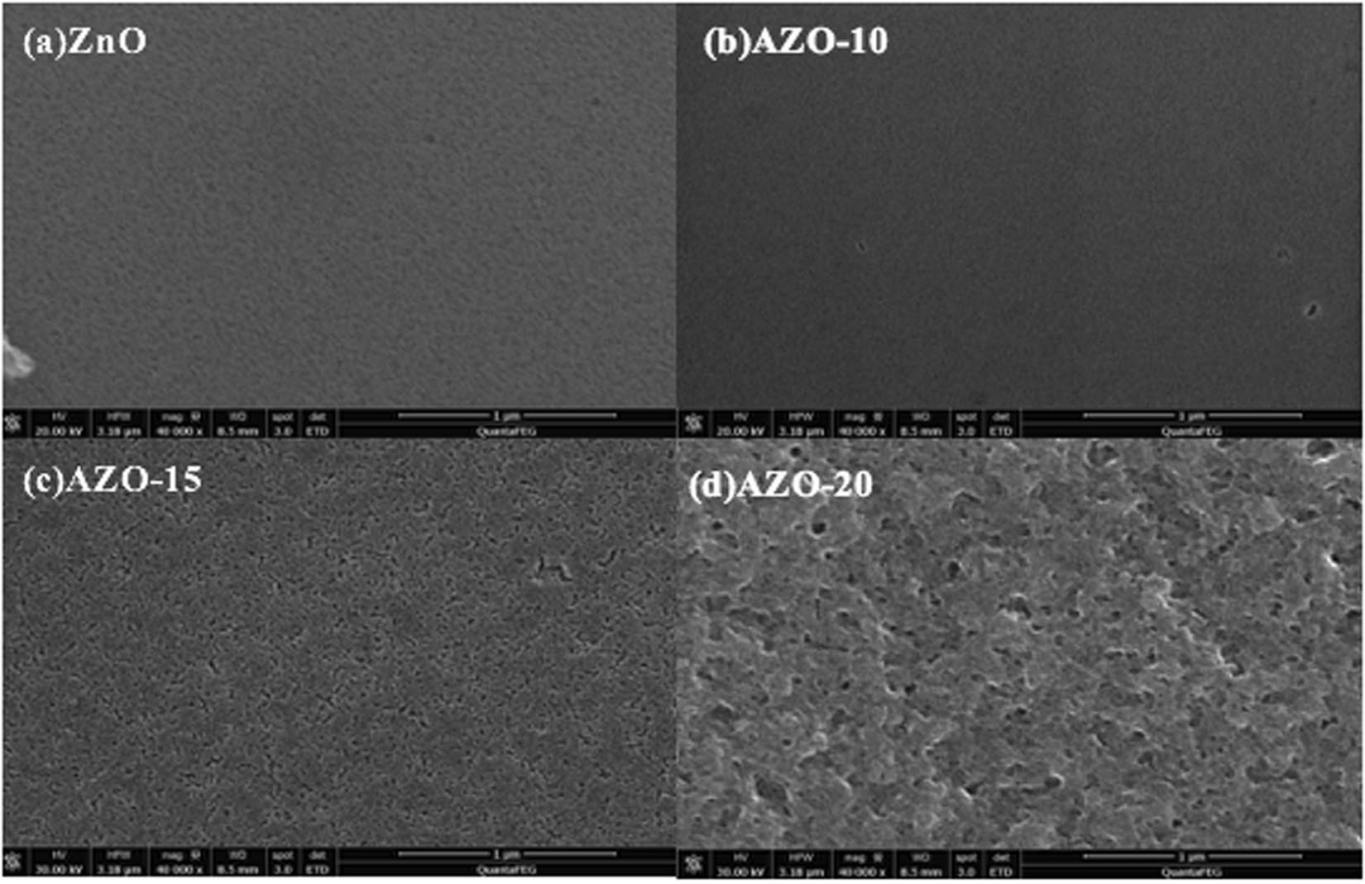
SEM image of ZnO, AZO-10, AZO-15 and AZO-20.

The PCE of devices with AZO do not get enhanced monotonously with doping concentration in AZO. Device performance is clearly compromised when using the AZO layer with higher Al doping concentration (>10%). Such an effect can be partially explained by the change of surface morphology. Shown in Fig. 6 are the scanning electron microscopy (SEM) images of the ZnO, AZO-10, AZO-15, and AZO-20 films on ITO substrates. Compared to the ZnO and AZO-10 films that have uniform and smooth surface, evenly-distributed pinholes are observed in the AZO-15 films and the AZO-20 film has even rougher surfaces with larger pinholes and defects. Therefore, the rough surfaces of the AZO layers with high Al doping concentrations (>10%) causes poor electrical contact between the AZO and active layers, thereby the charge collection, as well as the PCE. As summarized in Table 2, the device based on AZO-20 layer has a much lower Jsc and FF than the devices without AZO (FF = 57.0% vs 73.4% and Voc = 0.682 V vs 0.743 V).

**Table 2:** Photovoltaic performance parameters for the PTB7:PC_71_BM:ICBA devices with ZnO, AZO-2.5, AZO-5, AZO-10 and AZO-20 NPs as electron transport/selective layers.

ETLs	Voc (V)	FF (%)	Jsc (mA/cm^2^)	R at Voc (Ω)	R at Isc (Ω)	ηP (%)
ZnO	0.741 ± 0.001	73.4 ± 0.1	15.2 ± 0.3	101 ± 2	33986 ± 5	8.34 ± 0.1
AZO-2.5	0.742 ± 0.001	73.6 ± 0.1	15.3 ± 0.2	100 ± 3	34345 ± 5	8.41 ± 0.1
AZO-5	0.744 ± 0.001	72.9 ± 0.1	15.6 ± 0.1	102 ± 3	32294 ± 3	8.56 ± 0.2
AZO-10	0.743 ± 0.002	67.7 ± 0.3	14.9 ± 0.3	123 ± 2	17230 ± 8	7.56 ± 0.2
AZO-20	0.682 ± 0.002	70.0 ± 0.1	14.6 ± 0.3	213 ± 1	15199 ± 6	5.73 ± 0.3

**Figure 7:**
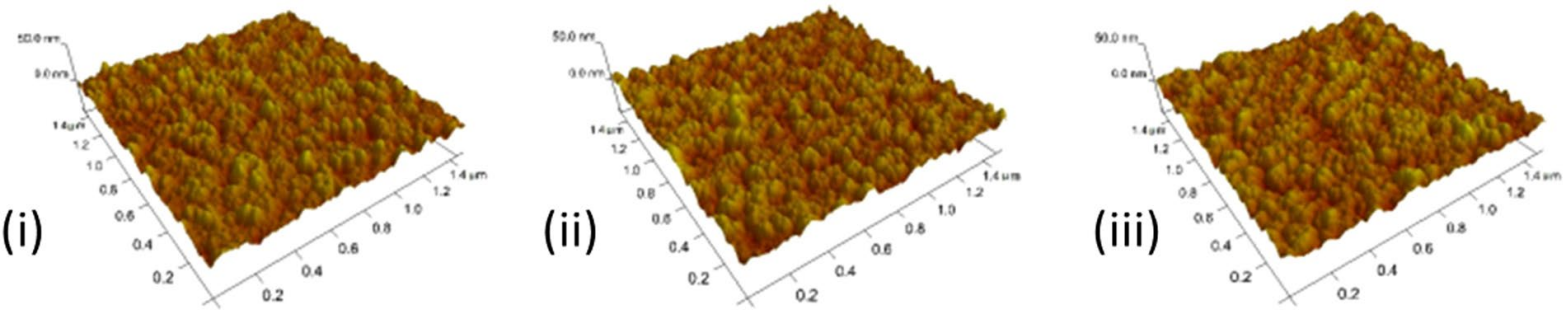
AFM topographic image of ZnO (AZO) Nanostructures film, (1.5 μm × 1.5 μm) (i) ZnO, 3.44 nm; (**ii**) AZO-10, 3.8 nm; (**iii**) ZnO/AZO-10, 3.63 nm.

The rough surfaces of AZO layers on ITO, especially for those with high Al doping concentrations (>10%), could be attributed to poor wetting of AZO on ITO. Therefore, the rough surface can be modified by adding a layer of ZnO NPs between AZO and ITO layers, which optimizes the surface energy. In addition, replacing part of AZO with ZnO reduces the optical penalty from the absorption of AZO^25^. For example, as shown in the atomic force microscopy images in Fig. 7, the surface roughness of a bilayer ZnO/AZO-10 is reduced to a comparable value of 3.63 nm as that of 3.44 nm for a ZnO layer, which is much lower than the pure AZO NP layer with the roughness of 3.8 nm. Moreover, once utilized such ZnO/AZO bilayer structure in the PTB7:PC_71_BM:ICBA system, an energy level buffering layer is formed between ITO and the active layer and thus to improve the electron transport/extraction efficiency.

**Figure 8:**
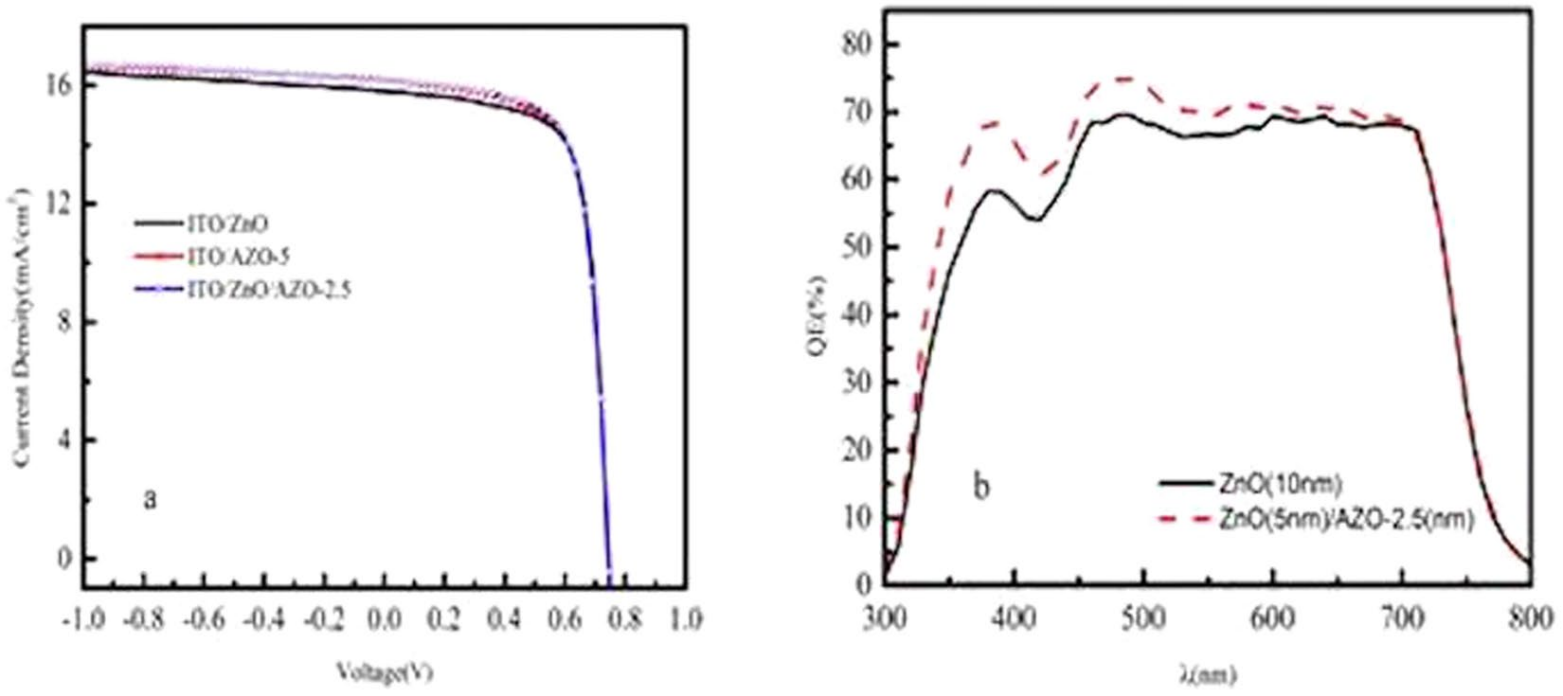
(**a**) The current density-voltage (J-V) charateristics measured under 1 sun ( = 100 mW/cm^2^) AM 1.5 G simulated solar illumination of device ITO/ETLs/P:P:I/MoO_3_/Ag. The three ETLs indicate ZnO(10 nm); AZO-5(10 nm); ZnO(5 nm)/AZO-2.5(5 nm); (**b**) The IPCE spectra of photovoltaic cells. ITO/ZnO (10 nm) /P:P:I /MoO_3_/Ag; ITO/ZnO (5 nm)/AZO-2.5 (5 nm)/P:P:I /MoO_3_/Ag.

**Figure 9:**
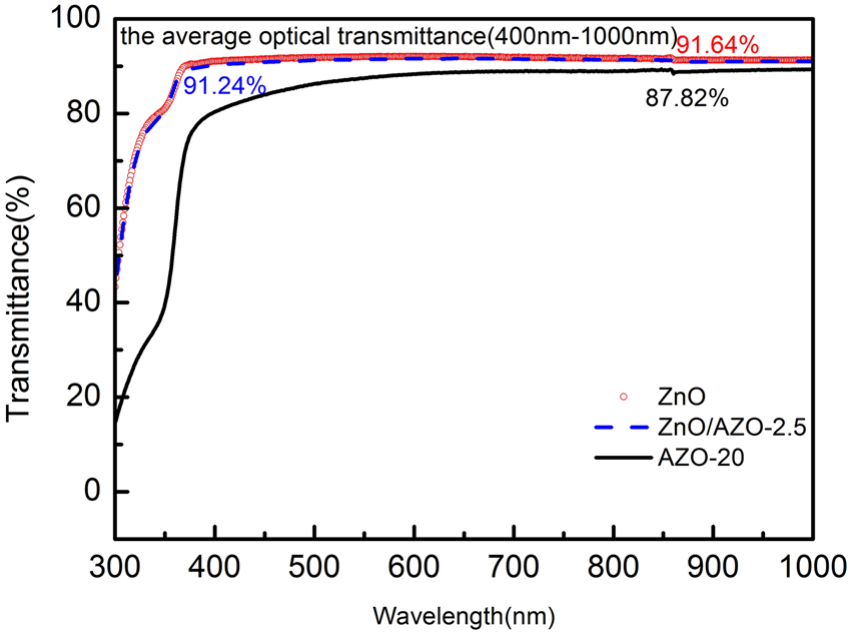
UV-visible transmittance spectra of glass/ZnO, glass/ZnO/AZO-2.5 and glass/AZO-20. The average optical transmittance form 400 nm- 1000 nm of glass/ZnO, glass/ZnO/AZO-2.5, and glass/AZO-20 are 91.64%, 91.24%, and 87.82%, respectively.

Figure 8(a) compares the J-V characteristics of the PTB7:PC_71_BM:ICBA devices with a single ZnO layer and ZnO/AZO bilayer as ETL under AM 1.5 G simulated solar illumination. Table 3 summarizes the photovoltaic performance parameters for the devices with a single ZnO transport layer and with ZnO/AZO bilayer with different Al doping concentrations. The devices based on ZnO/AZO bilayer show slightly higher Voc than those without AZO. Voc increases with increasing the Al doping concentrations in the AZO layers, which agrees with the argument of built-in potential. The FF of these devices is close to that of the ZnO based devices (FF = 73.4%), except that with ZnO/AZO-10 (FF = 72.2%), which can still be attributed to the relatively poor surface morphology. The majority of the improvements comes from Jsc. As shown in Fig. 8(a) and Table 3, the device based on the ZnO/AZO-2.5 layers has the highest Jsc of 15.8 mA/cm^2^, which is about 5% higher than that of the device based on the ZnO layer (Jsc = 15.2 mA/cm^2^). As shown in Fig. 8(b), incident photon-to-electron conversion efficiency (IPCE) as a function of the incident light wavelength for both ITO/ZnO and ITO/ZnO/AZO-2.5 devices were measured to identify the light loss in visible region. Overall, the device based on ZnO/AZO-2.5 bilayer shows about ~6% higher ηP the device without AZO (8.85% vs 8.34%). As mentioned above, doping-Al actually increases the electron mobility of the devices. But introducing metal Al might be a factor to reduce the transparency from ITO to active layer. In our work, compared to ZnO, there was obvious transmittance loss in the range of 400 nm-1000 nm when used AZO-20 as part of interlayer in one-layer ETL device, with the average optical transmittance of 91.64%, 87.82% as shown in Fig. 9. However, once utilized the bilayer structure of ZnO/AZO-2.5, transmittance is increased to 91.24% due to the modifizition of ZnO NPs as an optical spacer.

**Table 3:** Performance parameters of inverted organic solar cells using bilayer ZnO/AZO nanostructures.

ETLs	Voc (V)	FF (%)	Rat Voc(Ω)	Rat Isc(Ω)	Jsc(mA/cm^2^)	Eff(%)
ZnO	0.741 ± 0.001	73.4 ± 0.1	101 ± 2	33986 ± 5	15.2 ± 0.3	8.34 ± 0.1
ZnO/AZO-2.5	0.746 ± 0.001	73.4 ± 0.1	69 ± 1	33352 ± 6	15.8 ± 0.2	8.85 ± 0.2
ZnO/AZO-5	0.748 ± 0.002	73.3 ± 0.1	79 ± 1	37116 ± 5	15.1 ± 0.1	8.36 ± 0.1
ZnO/AZO-10	0.750 ± 0.001	72.2 ± 0.3	90 ± 2	26752 ± 7	15.5 ± 0.2	8.48 ± 0.1

To further enhance the built-in potential of the heterojunction, and thus achieve even better device performance. Here, we use a third transport layer, PFN^44^, to the ZnO/AZO structure. Conductive polymer PFN is a well-studied cathode modify material^35, 36^, which can form interface dipoles between the AZO and the photoactive layer(s) to improve the charge extraction efficiency. Surface potential of an AZO layer and PFN/AZO layer is measured by SKPM. As shown in (Figure S2) the surface potential of the AZO films increases by 130 mV after adding a thin layer of PFN, indicating a better energy level alignment with the active layers. As a result (shown in Table 4), using the ZnO/AZO/PFN ETL structure, simultaneous enhancement of Voc (from 0.745 V to 0.770 V) and Jsc (from 15.80 mA/cm^2^ to 16.37 mA/cm^2^) is further achieved, leading to a 5% improvement of PCE from 8.85% to 9.17% (The J-V and IPCE curves of the devices are shown in Figure S3).

**Table 4:** Photovoltaic performance parameters of the ITO/ETLs/P:P:I/MoO_3_/Ag with or without PFN as electron transport/selective layers.

ETL	Voc (V)	FF (%)	Jsc (mA/cm^2^)	ηP (%)
ZnO/AZO	0.745 ± 0.001	73.4 ± 0.1	15.80 ± 0.2	8.85 ± 0.2
ZnO/AZO/PFN	0.770 ± 0.003	72.1 ± 0.2	16.37 ± 0.2	9.17 ± 0.1

**Figure 10:**
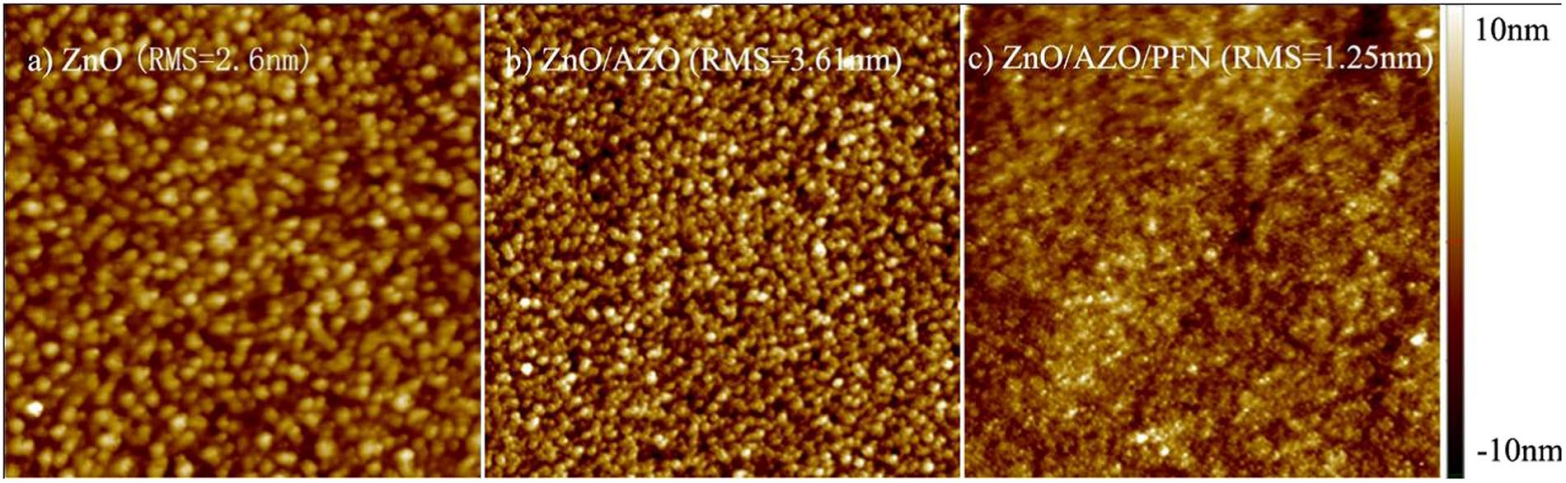
AFM topographic image, (5 μm × 5 μm) (**a**) ZnO, 2.6 nm; (**b**) ZnO/AZO, 3.61 nm; (**c**) ZnO/AZO/PFN, 1.25 nm.

Such enhancement can be first attributed to the improvement of film morphology the ETL stack. As shown in the AFM images in Fig. 10, the ZnO/AZO/PFN shows a much smoother surface (RMS = 1.25 nm) than that of the ZnO single layer and ZnO/AZO bilayer (RMS = 2.6 nm and 3.61 nm).

**Figure 11:**
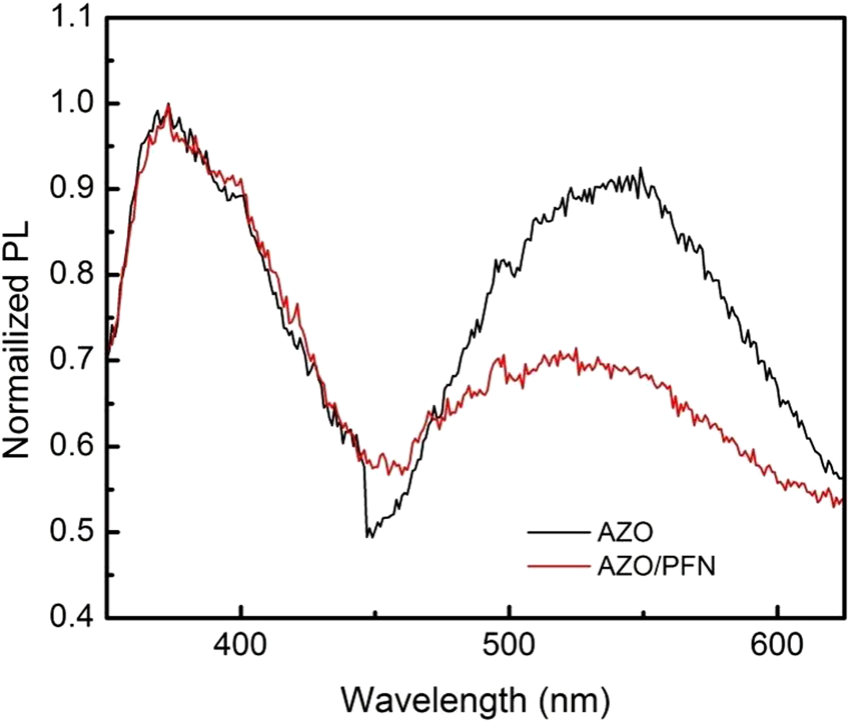
Normalized photoluminescence spectra. Black line represents the AZO layer, the red line represents the AZO/PFN layer.

Moreover, the addition of PFN is proved to effectively reduce the atomic defects at the AZO surface which can quench excitons and trap electrons. Compared in Fig. 11 is the normalized photoluminescence spectra of the AZO layer and AZO/PFN layer on glasses. The AZO NPs show one characteristic PL peak at 375 nm and one peak at 535 nm caused by the defects of AZO NPs^45^. The intensity of the defect emission is comparable to that of the intrinsic PL peak of the AZO NPs, indicating high concentration of defects in the AZO films. With a layer of PFN, the peak intensity of the defect based PL peak decreases to 70% of its origin value. Therefore, the surface defects of the AZO film can be partially passivated by the PFN layer, leading to a better device performance than that without PFN.

## Conclusions

Enhanced power conversion efficient was obtained when we utilized a two-layer structure of ZnO/AZO as ETL in ITO/ZnO/AZO-x/PTB7: PC_71_BM: ICBA/MoO_3_/Ag inverted devices in this work. Due to the low work function of optimized AZO nanoparticles and the resultant large built-in potential of the heterojunction, ZnO/AZO bilayer ETL effectively achieves more efficiency due to improve both the light transmittance of the device and the voltage as well as the charge transport of devices. PFN is used to further enhance the built-in potential of the heterojunction, and optimized devices with thiple-layer of ZnO/AZO/PFN show significant improvement on Jsc (16.37 mA/cm^2^), Voc (0.77 V), and PCE (9.17%).

## Experimental Details

### Fabrication of inverted PSCs

ZnO nanoparticles were synthesized by a solution-precipitation process using Znacetate and tetramethy-lammonium hydroxide (TMAH)^46^. For a typical synthesis, a solution of zinc acetate in dimethyl sulphoxide (DMSO) (0.5 M) and 30 ml of a solution of TMAH in ethanol (0.55 M) were mixed and stirred for 1 h in ambient air, then washed and dispersed in ethanol at a concentration of 30 mg/ml.

The devices were fabricated by spin-coating the ZnO(7.5 mg/ml) solution on the top of the pre-cleaned ITO glass, and the heated at 150 °C for 15 min in the air. Subsequently, AZO-x(7.5 mg/ml) was spin-coating on to the ZnO and heated at the same way. The PFN interlayer solution was prepared by dissolving PFN in methanol in the presence of a small amount of acetic acid (2 μl/ml) and its solution (concentration, 0.33 mg/ml) was spin-coated on top of the ZnO NPs layer with 5000 rpm for 30 s, then dried in vacuum at 80 °C for 5 min. Then, the samples were transferred into a nitrogen-filled glove box, and PTB7:PC_71_BM:ICBA (8:10.2:1.8) were dissolved in 1 ml of chlorobenzene with 3% DIO. The PTB7:PC_71_BM:IBCA blend was spin coated on top of the ZnO/AZO-x layer at 800 rpm for 120 s to form a BHJ layer. Subsequently, the samples were put into the pre-vacuumed at a pressure of approximately ~10^−2^ Pa for another 2 hours before being transferred into an evaporator. Finally, a 10 nm-thick MoO_3_ and 130 nm-thick Ag electrode was deposited by thermal evaporation at a pressure of approximately ~10^−4^ Pa. The active device area was 0.04 cm^2^ as defined by the patterned electrodes in a cross-bar geometry.

### Characterization and measurement

The topography of the ZnO (or AZO) film surface was evaluated by atomic force microscope (AFM, SPA400, Japan). Current density - voltage (J-V) characteristics of the solar cells in the dark and under simulated AM 1.5 G solar illumination were measured in laboratory ambient using a computer-programmed Keithley 2420 source/meter. The light intensity from the Xe-arc lamp solar simulator was calibrated using a single crystalline silicon reference cell. The IPCE measurements system (Enli Technology) comprised a xenon lamp, a monochromator, a chopper and a lock-in amplifier together with a calibrated silicon photodetector.

## Electronic supplementary material

Supplementary Information
