# Supplementary material for: Multiple electron transporting layers and their excellent properties based on organic solar cell
Source: Sci Rep. 2017 Aug 29;7:9571. doi: 10.1038/s41598-017-08613-7 (PMC5575332; doi:10.1038/s41598-017-08613-7)
Supplement: Supplementary file 1 — Supplementary Information [file 41598_2017_8613_MOESM1_ESM.pdf]

Supporting Information

Multiple electron transporting layers and their excellent properties based on organic solar cell

Ziyan Yang[1], Ting Zhang[1,*], Jingyu Li[1],Wei Xue[1], Changfeng Han[2],Yuanyuan Cheng[2], and Lei Qian[3]*
Weiran Cao[3], Yixing Yang[3], Song Chen[4]

Table S1 Under the 1000v test voltage(spacing 1cm), the resistance of ZnO , AZO-15 and AZO-20 was measured

n-layer	resistance
ZnO	11 Gohm
AZO-15	1013 Mohm
AZO-20	230 Mohm

Table S2 XPS result of Al2p content

At. %	ZnO	AZO-2.5	AZO-5	AZO-10	AZO-15	AZO-20
Al2p	0	1.211	1.058	1.843	1.886	2.097

Although the actual numbers of the Al concentration vary with XPS and XRD, it is confirmed that Al has been doped in ZnO nanocrystal and the trend of doping concentration matches with each other.

Table S3 XRD diffraction peaks comparison of ZnO and AZO

Plane	ZnO	AZO-5	Difference
100	31.79	31.24	—0.55
002	34.56	34.86	0.30
101	36.07	36.22	0.15

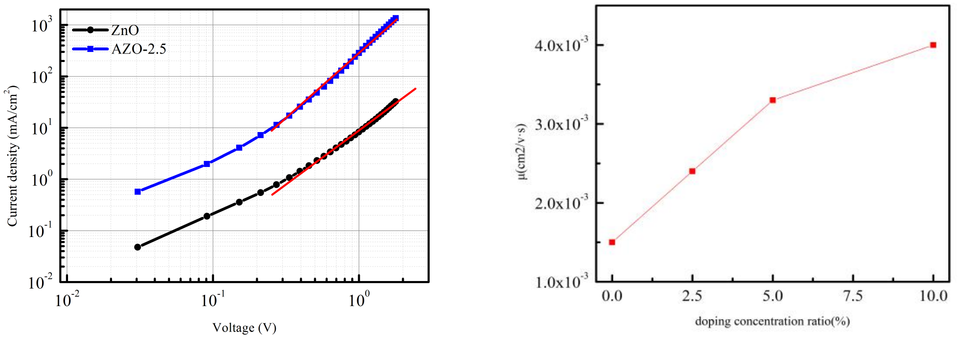

(a) **(b)**

Fig. S1 (a) Current-density – voltage (J – V) characteristic of an electron-only device with a structure of Al/ZnO NPs (400 nm)/Al and Al/AZO-2.5 NPs (400 nm)/Al. **(b)** electron mobility μ of 1.5 × 10^{-3} cm^2/V/s, 2.4 × 10^{-3} cm^2/V/s, 3.3 × 10^{-3} cm^2/V/s , 4.0 × 10^{-3} cm^2/V/s for ZnO, AZO-2.5, AZO-5, AZO-10, respectively.

Shown in **Figure S1 (a)** are the J-V characteristics of the electron-only devices (device structure: Al/ZnO (or AZO)/Al) based on ZnO and AZO-2.5. The electron mobility of the ZnO and AZO NP films is obtained by fitting the J-V characteristics in the space-charge limited current (SCLC) region ($J \propto V^2$) according to Mott-Gurney Law: $J = 9/8\,\varepsilon_0 \varepsilon_r \mu V^2 \bullet d^{-3}$, where ε_0 and ε_r are the relative and vacuum permittivity, and d is the film thickness. By assuming ε_r = 4,[1] we obtained a zero field electron mobility μ of 1.5 × 10-3 cm^2/V/s , 2.4 × 10^{-3} cm^2/V/s, 3.3 × 10^{-3} cm^2/V/s, and 4.0 × 10^{-3} cm^2/V/s, for ZnO, AZO-2.5, AZO-5, and AZO-10, respectively. (see **Figure S1 (b)**) The electron mobility of the ZnO layers increases with increasing the Al doping concentration. For example, the electron mobility of the AZO-5 film is more than two-fold of that of the pure ZnO layer, which improves the electron transport in the AZO layers and leads to an improved Jsc for the corresponding devices (15.6 mA/cm^2 vs. 15.2 mA/cm^2).

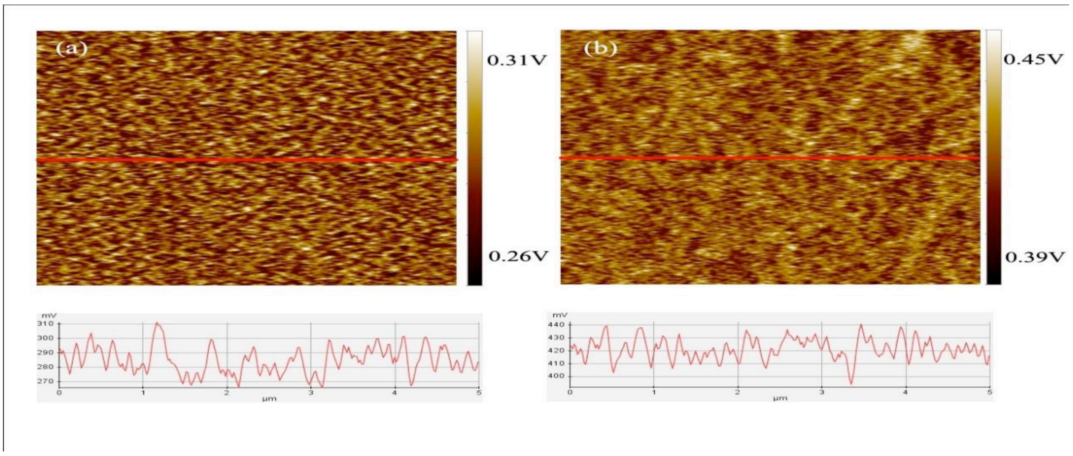

Fig. S2 Image of SKPM.

(a) AZO surface potential; **(b)** AZO/PFN bilayer surface potential

PFN can form dipole layer between AZO and the photo-active absorber layer which improves the surface potential, As shown in **Figure S2**, the surface potential of the AZO films increases by 130 mV after adding a thin layer of PFN. The interfacial dipole can improve the built-in electric field and improves electronic transmission. [2]

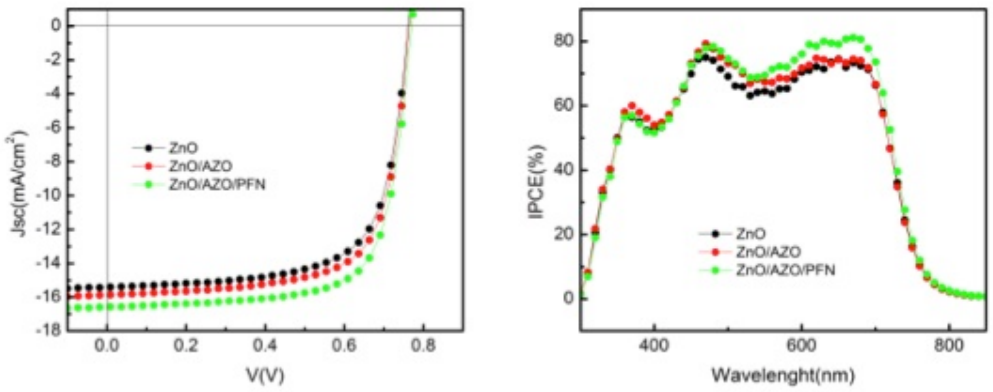

(a) (b)

Fig. S3 (a) Current-density – voltage (J – V) characteristic of the single, two-layer and triple-layer ETL devices (b) IPCE of the single, two-layer and triple-layer ETL devices

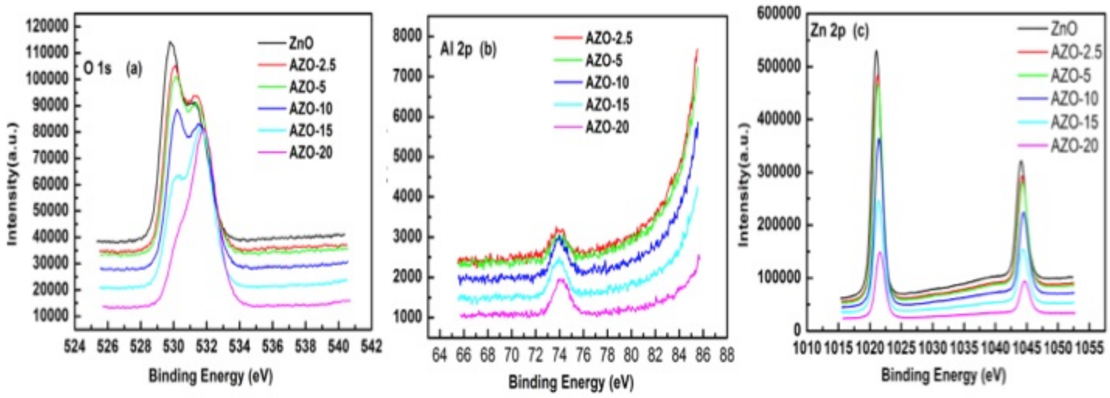

Fig.S4 XPS spectra corresponding to the (a) O 1s (b) Al 2p and (c) Zn 2p core levels of ZnO and AZO films

References

1 Li,W. Controlling molecular weight of a high efficiency donor-acceptor conjugated polymer and understanding its significant impact on photovoltaic properties, *Adv. Mater.* **26**, 4456-4462(2014).

2 Collins, B. A. Molecular miscibility of polymer-fullerene blends, *J. Phys. Chem Lett.* **1**, 3160-3166(2010).
